# Cooperative Reception of Multiple Satellite Downlinks

**DOI:** 10.3390/s22082856

**Published:** 2022-04-08

**Authors:** Haidar N. Al-Anbagi, Ivo Vertat

**Affiliations:** 1 Department of Electronics and Information Technology, Faculty of Electrical Engineering, University of West Bohemia, 30100 Pilsen, Czech Republic; ivertat@fel.zcu.cz; 2 Department of Communications Engineering, College of Engineering, University of Diyala, Diyala 32001, Iraq

**Keywords:** cooperative reception, diversity combining, receive diversity, SIMO, virtual ground station

## Abstract

Popular small satellites host individual sensors or sensor networks in space but require ground stations with directional antennas on rotators to download sensors’ data. Such ground stations can establish a single downlink communication with only one satellite at a time with high vulnerability to system outages when experiencing severe channel impairments or steering engine failures. To contribute to the area of improving the reception quality of small satellites signals, this paper presents a simple receive diversity scheme with proposed processing algorithms to virtually combine satellite downlink streams collected from multiple omnidirectional receivers. These algorithms process multiple received versions of the same signal from multiple geographically separated receiving sites to be combined in one virtual ground station. This virtual ground station helps detect the intended signal more reliably based only on a network of simple and cooperating software-defined radio receivers with omnidirectional antennas. The suggested receive diversity combining techniques can provide significant system performance improvement if compared to the performance of each individual receiving site. In addition, the probability of system outages is decreased even if one or more sites experience severe impairment consequences. Simulation results showed that the bit error rate (BER) of the combined stream is lower than the BER of the best quality receiving site if considered alone. Moreover, virtual ground stations with cooperative omnidirectional reception at geographically separated receivers also allow data to be received from multiple satellites in the same frequency band simultaneously, as software-defined receivers can digitize a wider portion of the frequency band. This can be a significant conceptual advantage as the number of small satellites transmitting data grows, and it is reasonable to avoid the corresponding necessary increase in the number of fully equipped ground stations with rotators.

## 1. Introduction

Small satellites are popular nowadays and mainly used for monitoring services. They can host sensors onboard or they can act as radio relays for in-space sensor networks. Collected sensors’ data is traditionally downloaded through single radio link between the small satellite and the related steerable ground station. This traditional single radio scheme is highly vulnerable to outages when experiencing severe channel degradations or when the steering engine unexpectedly fails. The other downside of such scheme is the inability to download data from more than one satellite simultaneously. Meanwhile, signal processing (SP) techniques have been widely developed to be deployed in wireless communications. One example of such SP involvements is diversity combining where multiple copies of the same signal are processed to exploit the spatial diversity gain [1]. Spatial diversity is one of the core mechanisms of the new generations of wireless systems to increase the capacity and reduce the bit error rate (BER) [2,3]. Such configuration would improve the overall wireless system performance by decreasing the probability of outages that each separate receiver may have because of some disturbances or any other impairment factors [4]. Spatial diversity can be configured at the transmitter, receiver, or even at both sides with multiple antenna arrangements [4]. Numerous publications on multiple-input multiple-output (MIMO) systems have been surveyed in [5] to provide a recapitulated document for newly interested researchers in such promising field.

This study, however, focused on the receive diversity technique and how it enhances the wireless system performance without any required modifications at the transmitter side.

To catch up with the requirements of new generations’ communications as well as the new technology trends, lots of research has been conducted to investigate and utilize the advantages of spatial diversity in general and receive diversity in specific. Receive diversity is a widely deployed antenna configuration in MIMO technology applications [6]. For instance, the navigation precision is improved through receive diversity combining to provide high accuracy location data to the autonomous driving cars [7]. Capable and professional ground stations can utilize a dual-channel global navigation satellite system (GNSS) receiver to receive dual-frequency signals simultaneously as presented in [8] to improve the location accuracy. It was shown in [9] that applying receive diversity combining at the analog feedback communication system (AFCS) improved the power bandwidth efficiency and reduced the detected errors. The same study suggested that such communication system with receive diversity would reach the required error threshold with less iterations compared to the single receiver system. To improve the system performance in the presence of multiple interferers, two models of receive diversity combining were presented in [10] to decrease the error rate. Furthermore, as the fifth generation (5G) wireless systems are promising for realizing modern and diverse smart cities applications, multiple orthogonal frequency division multiplexing (OFDM) versions of modulated imaged from monitored smart homes and smart industries were received and combined with maximal ratio combining (MRC) to improve the BER at the receiver [11]. Advanced SP methods have been utilized to enable the base station (BS) to pick the user with the highest signal to noise ratio (SNR) to stream out through beamforming [12,13]. In the area of energy harvesting, the receive diversity combining was suggested to increase the harvested RF energy by using three co-centric dipoles at the receiver [14]. Another receive diversity scheme was presented in [15] to increase the harvested RF energy using different combining techniques such as selection combining (SC) and MRC to harvest enough power for the wireless nodes enabling self-powering of these nodes. The simultaneous reception of two ionospheric-reflected signals in the range of (3–30) MHz could generate a SNR gain and hence improve the detection quality of the near vertical incidence skywave (NVIS) system as long as these two signals were uncorrelated with a correlation coefficient of less than 0.7 [16].

The receive diversity was successfully utilized to increase the transmission capacity of the optical modulator within its limited bandwidth in [17]. For reducing the outages probability in mobile networks, a receive diversity system was highly recommended rather than increasing the transmission power [18]. Due to timing error in OFDM systems, the BER can highly degrade the system performance. However, the performance of this system was significantly improved after deploying a receive diversity OFDM scheme instead [19]. The study conducted in [20] revealed that utilizing SIMO scheme for underwater sonar system enabled the distributed receivers to precisely locate the target despite the hostile underwater environment. A multi-reception technique also reflected remarkable system performance of the underwater optical communication (UWOC) system in [21] and the free space optical (FSO) communication in [22]. Based on the findings presented in [23], applying receive diversity with MRC on underwater acoustic (UWA) communication system mitigated the sever underwater multipath effects and reflected higher output SNR and lesser BER.

To the context of working in shadowed fading channel of inverse gamma, ref. [24] proposed a receive diversity combining to strengthen the SNR level at the receiver and increase the capacity of the system. To enhance the signal to interference (SIR) ratio of the wireless networks with large-scale coverage area, the authors of [25] considered multiple antennas at the receiver side. Moreover, single-input multiple-output (SIMO) system configuration was adopted in [26] to boost up the power of the received signal, mitigate the interference level at the receiver, and gain a network scaling. The outage probability at a receiver in a Poisson field of correlated interferes decreased according to [27] when exploiting multiple receiving antennas with MRC technique. To enhance the chaos-based radio communications, a SIMO architecture could be deployed especially for systems with problematic channel estimations [28]. Furthermore, a wireless communication system utilizing binary phase shift keying (BPSK) scheme was configured and tested in [29] under different scenarios where receive diversity combining outperformed other scenarios with the lowest BER value. SISO, 1 × 8 SIMO with MRC, and 1 × 8 multiuser MIMO (MU-MIMO) configurations were applied and examined on a wireless sensors network for their effectiveness in reducing sensors off probability in [30]. Out of these tested schemes, the 1 × 8 SIMO with MRC surpassed others in sustaining received signal-to-interference plus noise ratio (SINR) values of more than the threshold and therefore obtaining the least off probability. The quality of service (QoS) at mobile devices when receiving signals from satellites was investigated and tested for service continuity in [31] under two scenarios, with and without multi connectivity to 5G cellular networks. The multi-connectivity to a satellite and to cellular networks has ensured a reliable and continuous service at the mobile receiver, the investigation revealed.

Nowadays, the number of Internet of things (IoT) applications is exponentially growing, and hence recent research efforts have been more focused on maintaining reliable services through satellite communications [32]. In this context, this article proposes a receive diversity scheme based on the maximum likelihood with suggested SP algorithms as a basic study for the future implementation of omnidirectional ground stations network with continuous ad-hoc allocation of receiving sites along satellite trajectories to virtual ground stations. Multiple copies of the same transmitted signal are received by multiple geographically separated receivers. These multiple receivers cooperatively share these uncorrelated received versions of the transmitted signal with a virtual ground station to help detect the message signal more reliably. Doing so, even when one or more receiving sites are heavily affected by noise or other disturbances, there is still a possibility to retrieve the original signal from other cooperative sites with lower impairment levels. Hence, the outage probability of the whole system is significantly decreased. Therefore, the suggested network contributes mainly to the improvement of the small satellites’ downlink reception by reducing the BER, tracking more than a satellite at once, extending the communication window, and providing resistance against outages,

This paper is organized as follows: Section 2 describes the conventional single radio system and moves to the intended SIMO configuration with the suggested system model. The findings are presented and discussed in Section 3. Finally, Section 4 draws a brief conclusion.

## 2. System Description and Design

In communications, a system which involves a single radio link from the transmitter to the receiver is referred to as single-input single-output system (SISO). Conventional SISO satellite-to-ground communications are not efficient and reliable in providing guaranteed QoS coverage due to several reasons, one of which the inability to establish long time communication [33]. Limited time communication can be established only when a satellite passes over a ground station and hence no real time network status data can be collected for necessary dynamic changes [33]. When it comes to the QoS evaluation of digital receivers, some measures can be used to indicate the system performance and one of them is the rate of detected errors at the receiver, the measure which is known as the bit error rate (BER) [34,35]. BER is simply the number of the falsely detected bits to the total number of the transmitted bits during the observation time. Seeking as low BER as possible is the main objective of every digital communication link designer. However, different factors and challenges face the designing procedure as it involves a comprehensive study about the transmission channel, the surrounding environment, and receiver resources in addition to a compatible selection of a modulation scheme. Good consideration of these factors would consequently lead to a reliable radio communication link with low BER [36].

In this article, the process of receiving copies of the transmitted signal by diversity sites was simulated in MATLAB using binary frequency shift keying (BFSK)—one of the basic modulations used by radio-amateur satellites, and the signal quality was degraded using the additive white Gaussian noise (AWGN), a simple and powerful model in satellite communications. For comparison, BER calculation of both SISO (no diversity) and SIMO systems (with different algorithms of diversity combining) were realized.

To well execute the objective of this paper, we initially carried out the design of the conventional single communication link system and performed the BER calculation in the first part of this section. Then, we conducted multi-site cooperative system modeling and simulation in the following part of the section.

### 2.1. SISO System

**Figure 1:**
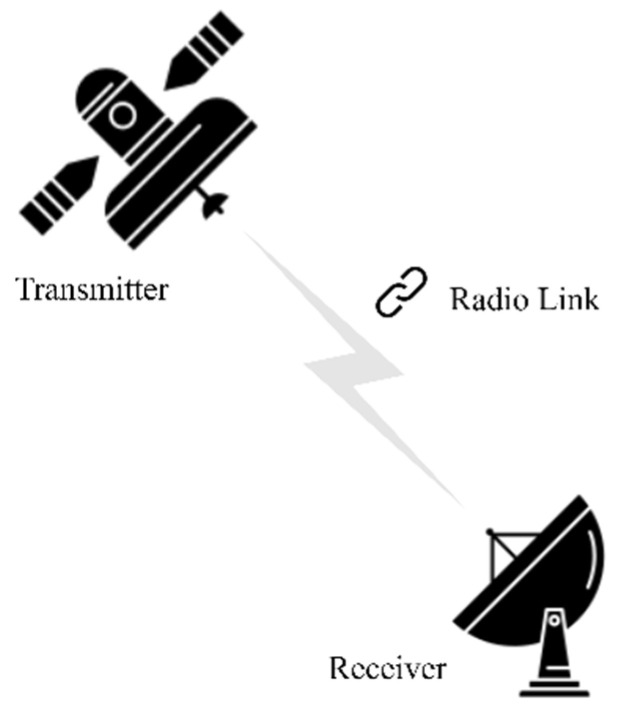
Single downlink communication.

**Figure 2:**
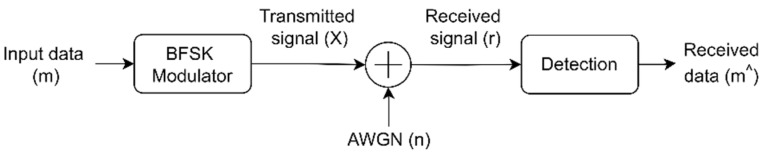
Block diagram of SISO system utilizing BFSK and considering AWGN channel.

Figure 1 simply visualizes the single radio link between the satellite as a transmitter and the ground station as a receiver. Such radio communication system utilizing a BFSK and considering an AWGN channel would be configured as in Figure 2.

The simulation starts with streaming the input bits, i.e., the message signal (*m*), in a unipolar form. Then, since BFSK is a two-dimensional modulation scheme which maps input data into orthogonal representations, an input (1) is mapped into (1) whereas input (0) is mapped into (*j*). These complex symbols are sent from the transmitter’s antenna to the receiver through the free space as a channel, which was a AWGN channel in our simulation. Through signal’s propagation, noise is added to the signal and since the transmitted signal is complex, the noise addition in the simulation is complex as well. Therefore, adding the complex noise (*n*) to the complex transmitted signal (*X*) represents the received signal (*r*) as shown in Equation (1):

(1) r=X+n

As mentioned earlier, the transmitted signal *X* has two representations and consequently, we have two states for the received complex signal as follows:

For input 1, the received signal is: r=((1+n)+(0+n) j) and similarly, for input 0, r=((0+n)+(1+n) j). That would create the detection hypothesis at the receiver to compare the real part to the imaginary part of the received signal and decide whether it is (1) if the real part is greater than the imaginary part and (0) if the other way around. The detection hypothesis is therefore:

(2) $$Real\;\left(r\right)\;\begin{array}{c} \begin{array}{c} 1\\ > \end{array}\\ \begin{array}{c} <\\ 0 \end{array} \end{array}\;Imag\left(r\right)$$

The theoretic BER of the BFSK system was calculated using the error function as well as the Q function to be plotted and compared to the simulation BER [37,38].

(3) $$\mathrm{BER}=\frac{1}{2}\;erfc\;\left(\sqrt{\frac{E_{b}}{2N_{o}}}\right)$$

(4) $$\mathrm{BER}=Q\;\left(\sqrt{\frac{E_{b}}{N_{o}}}\right)$$

**Figure 3:**
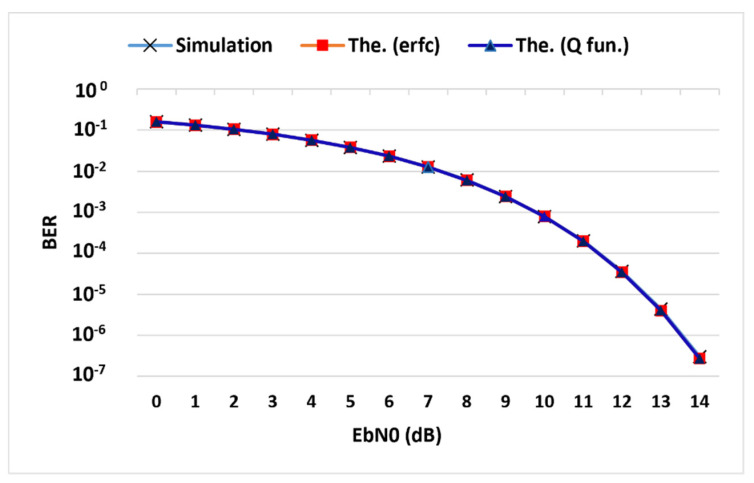
Simulation BER versus theory BER curves.

**Figure 4:**
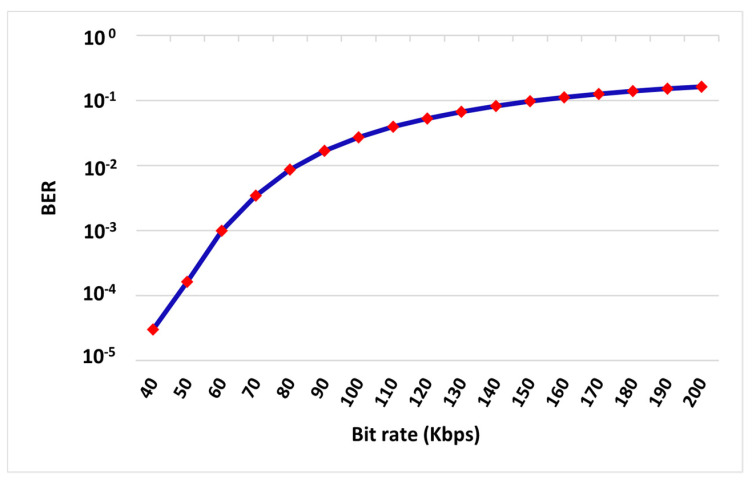
SISO BER versus data rate (at *E_b_/N_o_* = 10 dB).

The simulation and theory BER curves matched, as illustrated in Figure 3, with the same expected behavior where the system performance was improved when increasing the energy per bit. Moreover, the performance was further observed over different data rates at *E_b_/N_o_* of 10 dB and the resulting curve is presented in Figure 4.

Therefore, in order for SISO systems to function efficiently, we must either increase the transmitted power or to stream out at low signaling rates. However, that would be totally impractical and against the requirements of new generations’ communications of high data rate signaling and hostile environment serving. This simply summarizes the fact that SISO systems are not promising schemes to effectively fulfill the demands of the upcoming technology trends and thus alternative techniques are needed.

### 2.2. SIMO System

**Figure 5:**
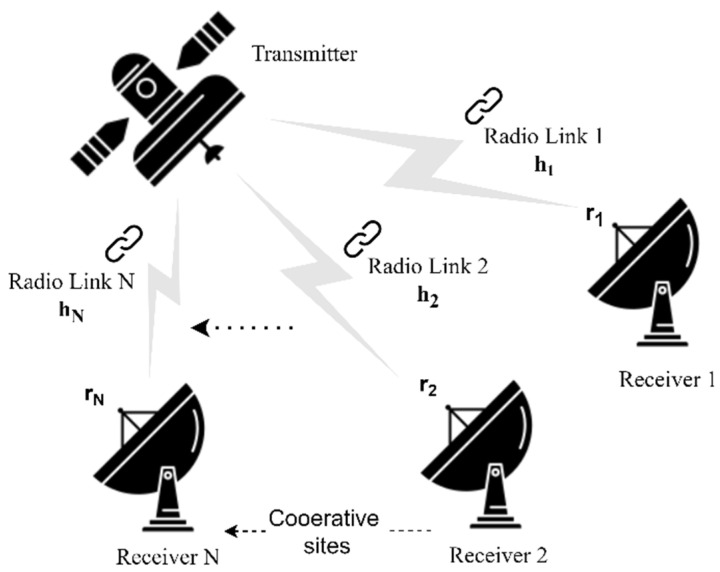
Multiple downlinks reception system.

The next step was to design the receive diversity system which involves the collection of multiple copies of the same signal by multiple receive antennas through multiple independent channels as illustrated in Figure 5.

**Figure 6:**
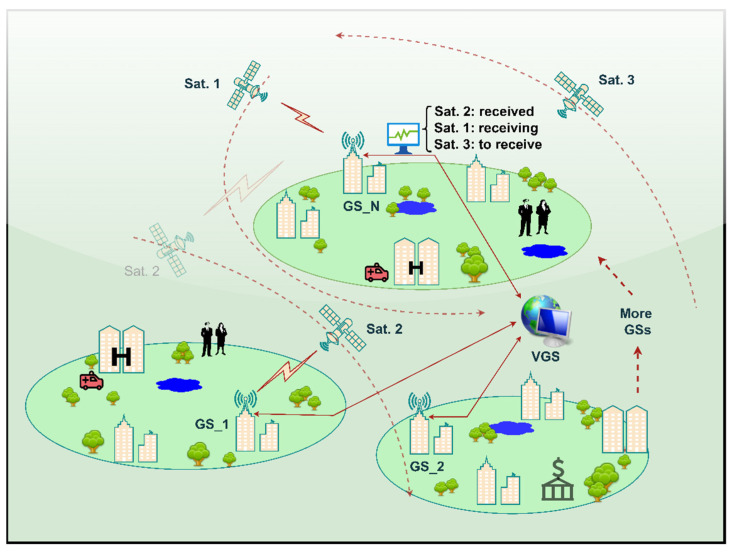
System model: cooperative omnidirectional ground stations (GS_1, GS_2, …, GS_N) along with the VGS in a network to receive data from multiple satellites simultaneously.

As Figure 5 demonstrates, the same message signal (*m*) is transmitted by the transmitter’s antenna and received by *N* receive antennas to collect *N* versions of the received signals (*r*_1_*, r*_2_ *… r_N_*) after being propagated through different and independent channels (*h*_1_*, h*_2_ *… h_N_*). These different paths would reflect different and independent impairment impacts on the same transmitted signal. Therefore, processing multiple copies of the same signal would help in case that one or more of these paths severely degrade the signal strength by constructing the message signal from the other good quality copies received by such a system. The received versions are processed and combined in a virtual ground station (VGS) to exploit the diversity gain, significantly improve the radio link quality, and decrease the outages probability. The aimed diversity gain can offer the opportunity to deploy the affordable omnidirectional antennas at the involved ground stations instead of the directional antennas with their expensive steering equipment. The lesser receiving antenna gain in this replacement would cause more detection errors which can be later compensated by the diversity gain. The proposed collaborative omnidirectional ground stations along with the virtual combining platform is modeled in Figure 6. This model shows how such network may track more than one satellite simultaneously, utilize omnidirectional receiving antennas, receive uncorrelated downlink replicas propagated through different independent paths, and provide resistance to outages.

In terms of system outages as a statistical quantity, (1, 2, …, *N*) earth stations operating in a single mode would have attenuations of *(A*_1_*(t)*, *A*_2_*(t)*, *…*, *A_N_(t))*, correspondingly. However, for these Earth stations to cooperatively work in a diversity mode, the cooperative receiving system would have a joint attenuation of *A_S_(t) = min [A*_1_*(t), A_2_(t), … A_N_(t)]*. Therefore, the attenuation of the system in a receiving diversity mode would be the minimum among all the cooperative Earth stations. Consequently, if one or two stations are deeply faded with high attenuation level, the receive diversity scheme would still be able to receive a good quality signal from other sites and share it with those attenuated stations [39]. Hence, the outage probability of the diversity mode versus single mode is framed as follows:

Assuming that the probability of outage in a single receiving site is *P_out_,* the probability of successful reception is (1 − *P_out_*). Suppose we engage *two* ground stations in the receive diversity system, then the system reception probability is:

- Success probability of (1 − *P_out_*)^2^, when both sites are receiving good quality signal;
- Success probability of (1 − *P_out_*)·(*P_out_*), when only one site is receiving good quality signal; and
- Outage probability of (*P_out_·P_out_*) = *P_out_*^2^, when both sites fail to receive good quality signal.

Accordingly, when engaging *N* Earth stations in the receive diversity system, the outage probability becomes (*P^N^)* which represents significant outages reduction compared to the conventional single-site systems.

In parallel, the system throughput, a QoS metric in satellite communications to indicate the number of received packets to the required processing time [40], can be maximized in the proposed scheme as the cooperative system receives *N* streams that can be simultaneously processed at the receiving sites.

After these motivational probabilistic concepts on how this receive diversity scheme can significantly reduce system outages and enhance the overall performance, the next step was to analyze the proposed system model.

Once the VGS receives the *N* downlink replicas with equal data length *L*, the combining matrix *C* is generated to eventually have the dimensions of *N*-by-*L*. Subsequently, the VGS triggers the combining algorithm to combine these multiple streams in a way so that the resulting combined stream, $r_{c},$ would have lesser BER than all BERs from the received versions. If we structure the combining matrix as:

$$C=\left[\begin{array}{ccc} r_{1}\left(1\right) & \cdots & r_{1}\left(L\right)\\ \vdots & \ddots & \vdots \\ r_{N}\left(1\right) & \cdots & r_{N}\left(L\right) \end{array}\right]$$

Then, instead of just selecting the stream $r_{n^{th}}$ out of the received versions based on the selection combiner (SC) method with respect to the criterion:

(5) $$\underset{n\in \left(1:N\right)}{min}\mathrm{BER}\;\left(r_{n^{th}}\right)$$

The aimed combined stream $r_{c}$ should achieve even lesser BER and it should be none of the received versions, i.e.,

(6) $$\underset{c\notin \left(1:N\right)}{min}\mathrm{BER}\;\left(r_{c}\right)$$

By interpreting the criterion in Equation (6) from the likelihood perspective, the combined stream’s likelihood, $L_{c}$, can be represented as:

(7) $$L_{c}\;(\;r_{c}|r_{1},\;r_{2},\;\ldots ,\;r_{N})$$

Since the received versions,$\;r_{1},\;r_{2},\;\ldots ,\;r_{N},$ are independent, the likelihood is therefore equivalent to the joint probability mass function (PMF) of these *N* versions and can be alternatively expressed as:

(8) $$\overset{N}{\underset{i=1}{\prod }}L_{c}\;(r_{c}|r_{i})$$

Equation (8) represents the likelihood quantity the VGS should maximize in the combining process to provide as good of an estimate of the transmitted stream as possible. To this context, one concept to reach this goal is to combine these streams with respect to the maximization of the likelihood function through the selection of the more likely occurring bit of each column in *C* to generate $r_{c}$ according to:

(9) $$r_{c}\left(k\right)=mode\;\left\{\;r_{1}\left(k\right),\;r_{2}\left(k\right),\;\ldots ,\;r_{N}\left(k\right)\right\}$$

where *mode* is the function that picks the most frequently occurring entry at each column in *C,* and *k* = 1, 2, …, *L*. If we assume that the original transmitted stream is $r_{0}$, the probability of detection errors can be expressed as [4]:

(10) $$P_{e}\left(k\right)=P\;\left(\;r_{c}\left(k\right)\neq \;r_{0}\left(k\right)\right)$$

In general, the minimization of the error probability $P_{e}$ corresponds to the maximization of the probability to provide a good estimate P to the originally transmitted data. Therefore, in our combining, the virtual ground station should generate a combined stream $r_{c}$ whose probability is

(11) $$P\;(\;r_{0}=r_{c}|r_{1:N},\;H)$$

where H denotes the channels impulse response matrix. The probability in Equation (11) can be further expressed using the conditional probabilities [4] as follows:

(12) $$P\;(\;r_{0}=r_{c}|r_{1:N},\;H)=\frac{P\;\left(r_{0}=r_{c}\right)f_{r_{1:N|r_{0},H}}(r_{1:N}|r_{0}=r_{c},\;H)}{f_{r_{1:N|H}}(r_{1:N}|H)}$$

where $f_{r_{1:N|H}}$ is the conditional probability density function (PDF) of $r_{1:N}$ given *H,* while $f_{r_{1:N|r_{0},H}}$ is the conditional PDF of $r_{1:N}$ given $r_{0}$ and *H*. Since the two terms $P\;\left(r_{0}=r_{c}\right)$ and $f_{r_{1:N|H}}(r_{1:N}|H)$ are independents of the aimed $r_{c}$, the maximization of the probability to generate good estimated combined stream is therefore reduced to maximizing only the dependent PDF term:

(13) $$f_{r_{1:N|r_{0},H}}\left(r_{1:N}|r_{0}=r_{c},\;H\right)$$

Thus, we can set our maximum likelihood (ML) goal in generating the combined stream as the combined stream as:

(14) $$r_{c_{ML}}=\underset{r_{c}\;\notin \;r_{1:N}}{argmax}\;f_{r_{1:N|r_{0},H}}\left(r_{1:N}|r_{0}=r_{c},\;H\right)$$

If we reform the conditional PDF term in Equation (14) using the general communication model of estimating the received signal in a MIMO system $r_{1:N}=H\;r_{0}+n_{1:N}$ [4], we obtain:

(15) $$f_{r_{1:N|r_{0},H}}\left(r_{1:N}|r_{0}=r_{c},\;H\right)=f_{n_{1:N}}\left(r_{1:N}-Hr_{c}\right)$$

where $f_{n}$ is the PDF of the white Gaussian noise (*n*), i.e., $f_{n}\left(n\right)=\frac{1}{{\left(\pi \sigma^{2}\right)}^{n}\;}e^{-\frac{1}{\sigma^{2}}{\left\|n\right\|}^{2}}.$ Accordingly, the left side of Equation (15) can be maximized by reducing ${\left\|n\right\|}^{2}$ for the aimed combined stream and hence the maximum likelihood can be achieved if the $\left(r_{1:N}-Hr_{c}\right)$ is minimized based on:

(16) $$r_{c_{ML}}=\underset{r_{c}\;\notin \;r_{1:N}}{argmin}{\left|\left|r_{1:N}-Hr_{c}\right|\right|}^{2}$$

To meet the criterion of distance reduction, i.e., the term ${\left|\left|r_{1:N}-Hr_{c}\right|\right|}^{2}$ in Equation (16), we applied the likelihood modal hypothesis on each column in the matrix *C* to generate the combined stream $r_{c}$. This combining can generate a stream which follows the tendency of the data towards the original data distribution. Hence, the combined stream can achieve lesser distance to the original stream than the distance from each received version to the original data. The proposed combining can be implemented by initially selecting the first received version out of the *N* received versions to be the pre-combined stream, $r_{pre\_c}$, i.e., $r_{pre\_c}=r_{1}.$ Then, the combined stream $r_{c}$ is derived from $r_{pre\_c}$ or its complement according to:

(17) $$r_{c}\left(k\right)=\{\begin{array}{ll} r_{pre_{c}}\left(k\right), & if\;mode\;\left\{\;r_{1}\left(k\right),\;r_{2}\left(k\right),\;\ldots ,\;r_{N}\left(k\right)\right\}=r_{pre_{c}}\left(k\right)\\ \bar{r_{pre_{c}}\left(k\right)}, & Otherwise \end{array}$$

Therefore, the combined stream can be derived from the received versions on a bit-by-bit inspection of the data tendency in all the received streams. This is promising to bring data into the combined stream that can better fit the original data distribution. In the results section, the Euclidean distance (ED) results of the $r_{0}$-to-$r_{c}$ and $r_{0}$-to-$r_{1:N}$ can verify how much reduction in that distance this algorithm can provide.

The MATLAB simulation of the proposed SIMO system can be configured from multiple SISO links whose simulation steps are detailed in Section 2.1. These SISO links were degraded with different impairment levels to create independent and uncorrelated received versions of the same transmitted data. Therefore, the simulation started with generating random unipolar data and then mapping these input bits into complex BFSK symbols. In the next step, *N* complex AWGN streams were generated and added to form *N* multiple uncorrelated versions of the received signal to be fed to the detector. The detection process passed the detected bit stream from each received version to the VGS to establish the combining square matrix *C* where the *n*^th^ row corresponds to detected bits from the *n*^th^ receiving site. The VGS hence started applying the maximum likelihood hypothesis expressed in Equation (17) on that square matrix based on vertical bit disagreement rule. This maximum likelihood detection was performed at the combining matrix in an ascending tempo starting from the 1st row to the *N*th one representing the possibilities of combining multiple streams gradually. Therefore, the first case represents conventional single stream detection as it performed detection on the first row only. The following step was for two sites combining and so on until the last trial was for combining all the *N* receiving sites together. This helped visualize the system BER improvement when gradually increasing the number of cooperative sites. The simulation was performed on a million-bit data stream received by 14 uncorrelated sites with *SNRs* of (0–13) dB.

## 3. Results and Discussion

**Figure 7:**
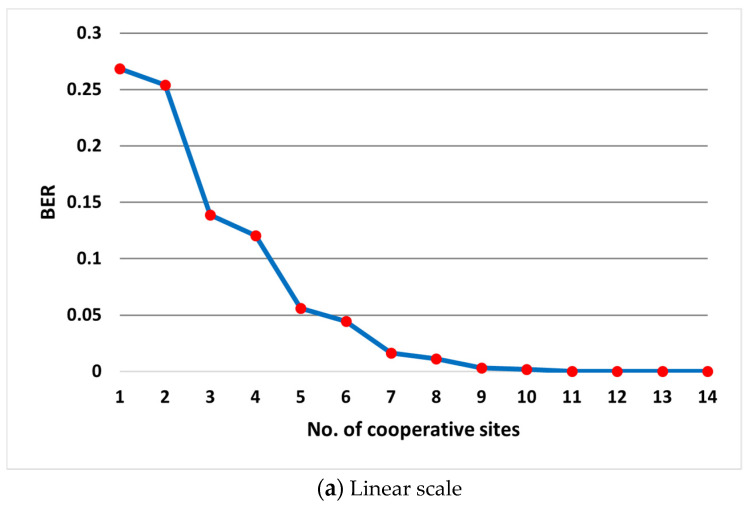

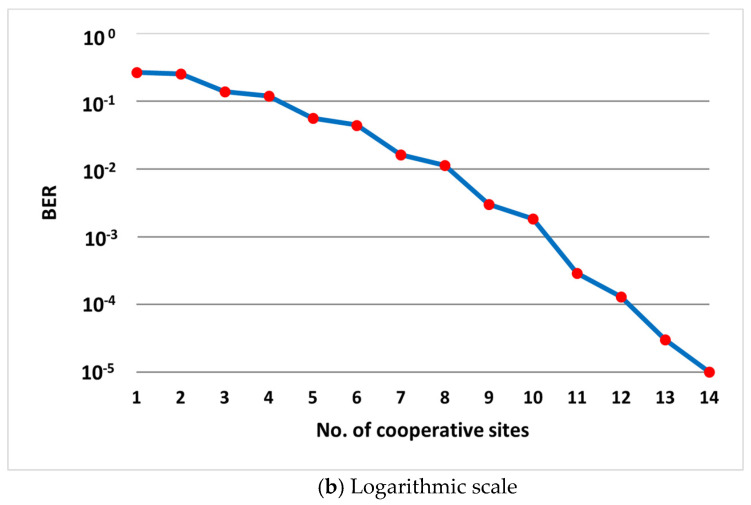
BER receive diversity combining of 14 sites with SNRs ranging between (0–13) dB: (**a**) linear scale, (**b**) logarithmic scale.

The BER results of the combining trials are presented in Figure 7. It is clear to see that the BER improved as we combined more receiving sites. Interesting behavior in the combining trials was that the BER of the system slightly improved when observing two successive trials from an odd number of sites to an even number. On the other hand, there was significant BER improvement between two successive trials when the first trial was for an even number of sites and the following trial was odd. This behavior was due to the fact that the detection hypothesis was based on the bit disagreement rule which has more errors likely to occur when the number of entrees is even. To enhance the performance of the system even more and to overcome this drawback, couple of SP techniques were proposed and examined for optional further improvement. So far, the presented combining algorithm neither required quality metrics evaluations (e.g., SNR) nor involved any weighting. However, the following suggested further improvement techniques would require metrics evaluation, but no weighting would be needed.

### 3.1. Exclusion of the Worst Stream

**Figure 8:**
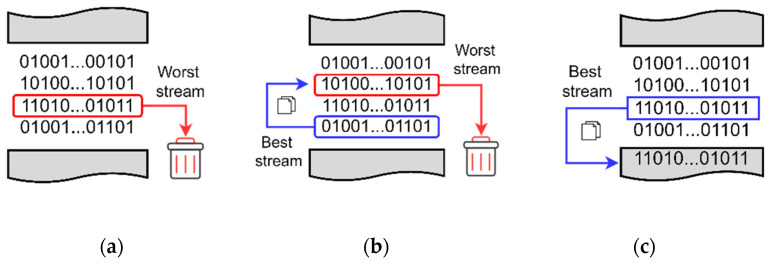
Illustrations of the SP algorithms: (**a**) worst excluded, (**b**) best replacing the worst, (**c**) best always tailed.

Exclusion of the worst stream was the first technique to improve the performance. This technique could be implemented after calculating the BER of all received versions and then deciding the stream with the highest BER value to be the worst quality stream. Then, the VGS formed the combining matrix *C* from all versions but the worst one, as illustrated in Figure 8a, and consequently the combining process was done without the worst stream which was holding the system’s performance back by introducing more possible errors to the combined stream than the rest streams. Doing so, the detection weight of the better-quality streams increased while nulling the detection weight of the worst stream.

### 3.2. The Best Stream Replacing the Worst One

The best stream replacing the worst stream could be also implemented to improve the results even more. After calculating the BER of all received streams, the highest BER stream was replaced with the lowest BER stream (i.e., the best quality stream replacing the worst quality stream as demonstrated in Figure 8b). Eventually, the combining algorithm combined all versions based on doubled detection weight for the best quality stream and no weight for the worst version which improved the results even further.

### 3.3. The Best Stream Is Repeatedly Injected

The third optional technique that could be implemented in the proposed cooperative system is when the best stream is repeatedly injected. Out of the received versions, the one with the lowest BER could be repeatedly tailed to the combining matrix, as visualized in Figure 8c, at each combining trial. This could gradually impose more detection weight to the best stream and hence achieve less errors at the combined stream.

The improvement techniques can be modeled the same as the raw combining in Equations (16) and (17) except the required pre-combining procedures of each technique. For instance, in the first technique, the worst stream should be excluded out of the combining matrix and then the same modeling applied for combining the new (*N* − 1)-by-*L* combining matrix *C*, as demonstrated earlier, to get the following ML criteria:

(18) $$r_{c_{ML}}=\underset{r_{c}\;\notin \;r_{1:N-1}}{argmin}{\left|\left|r_{1:N-1}-Hr_{c}\right|\right|}^{2}$$

where the initial assumption of $r_{pre\_c}=r_{1}$ can be also valid then modified to get the $r_{c}$ based on:

(19) $$r_{c}\left(k\right)=\{\begin{array}{ll} r_{pre_{c}}\left(k\right), & if\;mode\;\left\{\;r_{1}\left(k\right),\;r_{2}\left(k\right),\;\ldots ,\;r_{N-1}\left(k\right)\right\}=r_{pre_{c}}\left(k\right)\\ \bar{r_{pre_{c}}\left(k\right)}, & Otherwise \end{array}$$

Table 1 lists the achieved BER of each suggested SP technique versus the raw combining BER values. Excluding the worst stream out of the combined streams reflected slight improvement compared to the original combining BER values. Replacing that worst stream with the best stream improved the performance even more. The best achieved BER values were when always injecting the best stream into the combining matrix which reached zero error at six receiving sites combining.

**Figure 9:**
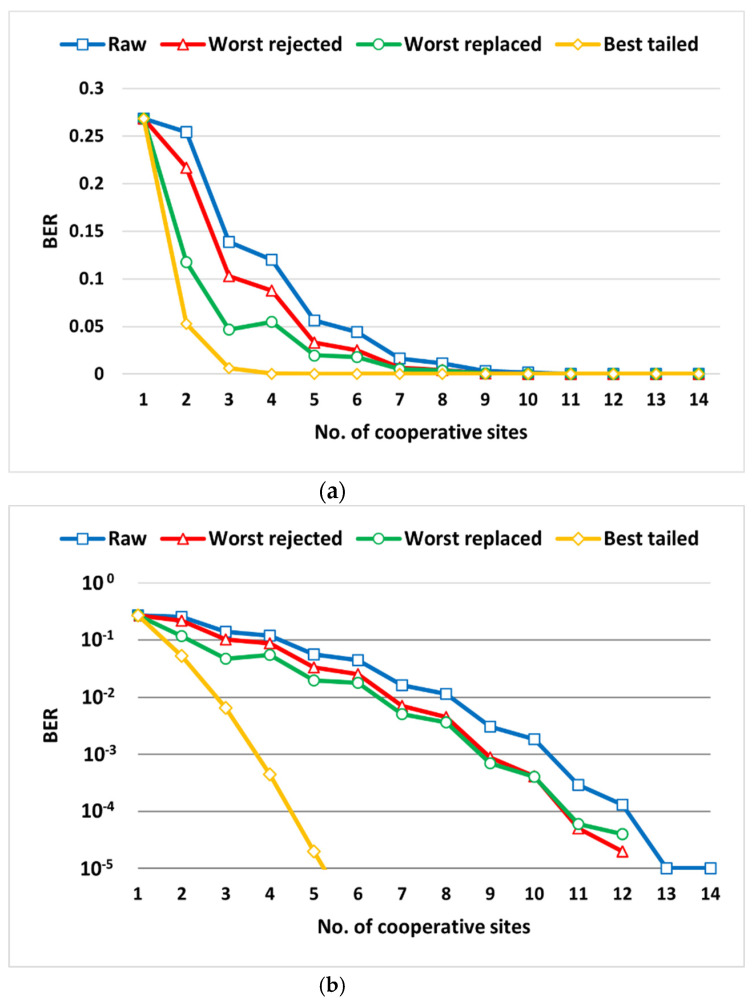
BER curves from raw and the proposed SP algorithms: (**a**) linear, (**b**) logarithmic.

Figure 9 presents the simulation BER results of the original combining algorithm as well as the three proposed algorithms in linear and logarithmic scales.

The three SP algorithms improved the system performance in achieving less detected errors compared to the raw combining. Rejecting the worst quality stream slightly enhanced the system performance with more than 50% errors reduction in the combining trials of more than seven sites. Replacing the worst stream with the best stream led to even better performance. In the last proposed technique, the best stream was repeatedly tailed to the combining matrix, imposing more detection weight for the least corrupted received signal version. Doing so, the system achieved zero errors detection as early as when only six sites were engaged. Figure 9b does not show that achieved zero BER due to the infinitive logarithmic quantities which explains why linear scale curves are also provided.

The Euclidean distance (ED) can be measured to indicate how close the received versions as well as the combined one are from the original message stream through:

$$ED_{a,b}=sqrt\left(\left(a-b\right)*{\left(a-b\right)}^{\prime }\right)$$

where a and b can be any signal vectors of the same length. In a million-bit length of data streams, the least calculated ED between the message and the received versions $r_{1:N}$ was 18.0831, whereas it was only 3.1623 between the message signal and the combined stream $r_{c}$. This reflects how alike the combined stream and the original message are and how well the combined stream approached the original data distribution.

The proposed improvement algorithms were tested for consistency to different data sample sizes and showed great scalability by reflecting the same system behavior. Table 2 summarizes the elapsed execution times for the 1-milion data set used in this study in addition to other different data lengths.

## 4. Conclusions

In conclusion, the traditional satellite-to-ground station communication system is highly vulnerable to outages and not in favor of the new generations of wireless communications trends since its performance is significantly degradable when the data rate is high and the received signals are deeply faded. In addition, a ground station in such single radio link can only track one satellite at a time through steerable high-gain antenna. The unexpected mechanical failures of the antenna rotator then create another cause of outages.

To avoid the consequences of such circumstances, the receive diversity combining was recommended to significantly improve the satellite downlink reception quality. This work proposed a network of ground stations with affordable omnidirectional antennas instead of the high-gain antennas to cooperatively receive multiple replicas of the same downlink signal to be demodulated and then combined. Combining multiple independent and uncorrelated copies of a satellite downlink signal based on maximum likelihood helped reduce detection errors. Deploying the proposed cooperative reception scheme on 14 separate omnidirectional ground stations created sufficient diversity gain to compensate the lesser receiving antenna gain and hence achieved significant reduction in the BER for the combined stream. The combined stream achieved lesser ED to the original data if compared to all other received versions. The suggested combining algorithm neither requires SNR calculations nor involves weightings. However, additional three improvement techniques that require quality metrics were examined and can be utilized for further improvement. Excluding the worst stream in the combining matrix reflected better system performance. Replacing the worst stream with the best stream imposed more weight of the less corrupted signal version and consequently performed even better. The best algorithm was when always tailing the best candidate into the combining matrix which significantly improved the system performance, achieving zero detected errors at the six sites combining trial. The algorithms were tested against different data lengths and showed great scalability with reasonable processing elapsed times.

The presented cooperative reception scheme of the omnidirectional ground stations along with the virtual combining platform can achieve lower BER with less required *E_b_*/*N_o_*, track more than a satellite at once, extend the communication window, provide resistance against outages, exploit the available online open source SDR networks for streaming the data out through the Internet, and offer to replace the expensive directive antennas and their steering engines with much affordable omnidirectional antennas.

## Figures and Tables

**Table 1:** BER results from raw receive diversity and proposed SP algorithms.

No. of Sites	Raw	Worst Excluded	Worst Replaced	Best Tailed
1	0.26862	0.26862	0.26862	0.26862
2	0.25414	0.21686	0.11775	0.05261
3	0.13884	0.10295	0.0468	0.00645
4	0.12037	0.08797	0.05483	0.00044
5	0.05619	0.03321	0.01966	2 × 10^−5^
6	0.04439	0.025	0.01785	0
7	0.01624	0.00694	0.00501	0
8	0.01131	0.00445	0.00362	0
9	0.00301	0.00087	0.00069	0
10	0.00183	0.00041	0.0004	0
11	0.00029	5 × 10^−5^	6 × 10^−5^	0
12	0.00013	2 × 10^−5^	4 × 10^−5^	0
13	3 × 10^−5^	0	0	0
14	1 × 10^−5^	0	0	0

**Table 2:** Elapsed time in (sec.) taken by the algorithms to combine 14 streams.

Data Length	Worst Excluded	Worst Replaced	Best Tailed
1 × 10^6 1^	9.439559	10.967350	19.539677
1 × 10^5^	0.948979	1.131833	2.061516
1 × 10^4^	0.108183	0.130703	0.256498
1 × 10^3^	0.043260	0.050263	0.056976
2 × 10^6^	18.795995	21.877629	38.563990

^1^ Denotes the used data length in this study.

## Data Availability

Not applicable.

## References

[1] Kim M., Park D.J. (2020). Learnable MIMO detection networks based on inexact ADMM. IEEE Trans. Wirel. Commun..

[2] Lv Y., He Z., Rong Y.J. (2020). Two-way AF MIMO multi-relay system design using MMSE-DFE techniques. IEEE Trans. Wirel. Commun..

[3] Sun W.-B., Tao M.-L., Wang L., Yang X., Zhou R.-Z., Yang Z.-X. (2021). Joint Resource Allocation for Multiuser Opportunistic Beamforming Systems with OFDM-NOMA. Entropy.

[4] Proakis J.G., Salehi M. (2001). Digital Communications.

[5] Mietzner J., Schober R., Lampe L., Gerstacker W.H., Hoeher P.A. (2009). Multiple-Antenna Techniques for Wireless Communications—A Comprehensive Literature Survey. IEEE Commun. Surv. Tut..

[6] Kelechi A.H., Alsharif M.H., Oluwole D.A., Achimugu P., Ubadike O., Nebhen J., Aaron-Anthony A., Uthansakul P.J.S. (2021). The Recent Advancement in Unmanned Aerial Vehicle Tracking Antenna: A Review. Sensors.

[7] Matthie S., Senega S., Lindenmeier S. An Antenna Diversity and Combining System for Improved Mobile GNSS Reception. Proceedings of the 2019 49th European Microwave Conference (EuMC).

[8] Qi N., Xu Y., Chi B.Y., Xu Y., Yu X.B., Zhang X., Xu N., Chiang P., Rhee W., Wang Z.H. (2012). A Dual-Channel Compass/GPS/GLONASS/Galileo Reconfigurable GNSS Receiver in 65 nm CMOS with On-Chip I/Q Calibration. IEEE Trans. Circuits Syst. I Regul. Pap..

[9] Tengshe R., Kumar N. Receive Diversity in Analog Feedback Communication. Proceedings of the 2019 PhD Colloquium on Ethically Driven Innovation and Technology for Society (PhD EDITS).

[10] Aruna G., Barman M.P. Performance Analysis of Advanced Diversity Receivers in the Presence of Multiple Interferers. Proceedings of the 2018 International Conference on Wireless Communications, Signal Processing and Networking (WiSPNET).

[11] Kansal L., Gaba G.S., Chilamkurti N., Kim B.G. (2021). Efficient and Robust Image Communication Techniques for 5G Applications in Smart Cities. Energies.

[12] Van Le T., Lee K.J. (2020). Adaptive perturbation-aided opportunistic hybrid beamforming for mmWave systems. IEEE Trans. Veh. Technol..

[13] Yu Q., Han C., Bai L., Choi J., Shen X.J. (2018). Low-complexity multiuser detection in millimeter-wave systems based on opportunistic hybrid beamforming. IEEE Trans. Veh. Technol..

[14] Rezazadeh N., Shafai L. A pattern diversity antenna for ambient RF energy harvesting in multipath environments. Proceedings of the 2018 18th International Symposium on Antenna Technology and Applied Electromagnetics (ANTEM).

[15] Altinel D., Kurt G.K. Diversity combining for RF energy harvesting. Proceedings of the 2017 IEEE 85th Vehicular Technology Conference (VTC Spring).

[16] Male J., Porte J., Gonzalez T., Maso J.M., Pijoan J.L., Badia D. (2021). Analysis of the Ordinary and Extraordinary Ionospheric Modes for NVIS Digital Communications Channels. Sensors.

[17] Jung S.-Y., Kim C.-H., Park H.J., Ha I., Han S.-K. (2018). Receive diversity-based SNR improvement in OPDM-OFDMA-PON single-wavelength multiple access. J. Lightwave Technol..

[18] Das M., Sahu B. Effect of MRC diversity on outage probability in mobile networks. Proceedings of the 2019 Global Conference for Advancement in Technology (GCAT).

[19] Harun-Owr-Roshid M., Majumder S. Performance evaluation of a SIMO-OFDM wireless communication system impaired by timing error. Proceedings of the International Conference on Electrical & Computer Engineering (ICECE 2010).

[20] He C.F., Wang Y.Y., Yu W.B., Song L. (2019). Underwater Target Localization and Synchronization for a Distributed SIMO Sonar with an Isogradient SSP and Uncertainties in Receiver Locations. Sensors.

[21] Nath N., Anees S. Performance Analysis of SIMO-UWOC System. Proceedings of the 2020 IEEE International Conference on Advanced Networks and Telecommunications Systems (ANTS).

[22] Zheng A., Huang Y., Gao S.J. (2021). Modeling and Spatial Diversity-Based Receiving Improvement of In-Flight UAV FSO Communication Links. Appl. Sci..

[23] Zhao A.B., Zeng C.G., Hui J., Wang K.R., Tang K.Y. (2021). Study on Time Reversal Maximum Ratio Combining in Underwater Acoustic Communications. Appl. Sci..

[24] Upaddhyay V.K., Chauhan P.S., Soni S.K. (2021). Effective capacity analysis of SIMO system with MRC and SC over Inverse-Gamma shadowing. Int. J. Electron..

[25] Jiang D., Cui Y.J. (2019). Enhancing performance of random caching in large-scale wireless networks with multiple receive antennas. IEEE Trans. Wirel. Commun..

[26] Lee N., Baccelli F., Heath R.W. (2016). Spectral efficiency scaling laws in dense random wireless networks with multiple receive antennas. IEEE Trans. Inf. Theory.

[27] Wang Y., Liu F., Wang C., Wang P., Ji Y.J. (2020). Outage probability of SIMO MRC receivers with correlated Poisson field of interferers. IEEE Commun. Lett..

[28] Mobini M., Kaddoum G., Herceg M. (2022). Design of a SIMO Deep Learning-Based Chaos Shift Keying (DLCSK) Communication System. Sensors.

[29] Jethi G.S., Belwal N., Sunori S., Juneja P. Improvement in BER performance of BPSK system using diversity techniques. Proceedings of the 2017 International Conference on Computing, Communication and Automation (ICCCA).

[30] Yu Y., Mroueh L., Martins P., Vivier G., Terre M. (2020). Radio Resource Dimensioning for Low Delay Access in Licensed OFDMA IoT Networks. Sensors.

[31] Chung H., Kim J., Noh G., Won S.H., Choi T., Kim I. Demonstration of service continuity based on multi-connectivity with cellular and satellite access networks. Proceedings of the 2021 International Conference on Information and Communication Technology Convergence (ICTC).

[32] Munari A., Clazzer F.J. (2021). Spectral Coexistence of QoS-Constrained and IoT Traffic in Satellite Systems. Sensors.

[33] Li T., Zhou H., Luo H., Yu S.J. (2017). SERvICE: A software defined framework for integrated space-terrestrial satellite communication. IEEE Trans. Mob. Comput..

[34] Kumar G.K., Tejaswini K.S., Prashanthi U., Gayathri T. Maximized Spectral Efficiency and QoS Based Power Allocation Schemes for Co-operative UWB-WBAN. Proceedings of the 2019 IEEE International Conference on Electronics, Computing and Communication Technologies (CONECCT).

[35] Same M.H., Gleeton G., Gandubert G., Ivanov P., Landry R. (2021). Multiple Narrowband Interferences Characterization, Detection and Mitigation Using Simplified Welch Algorithm and Notch Filtering. Appl. Sci..

[36] Sklar B.J. (2001). Digital Communications Fundamentals and Applications.

[37] Zaidi M., Bouazzi I., Usman M., Shamim M.Z. Virtual Prototype of a wireless sensor node using VHDL-AMS. Proceedings of the 2020 Fourth World Conference on Smart Trends in Systems, Security and Sustainability (WorldS4).

[38] Nikitin P., Normark E., Wakayama C., Shi R. VHDL-AMS modeling and simulation of BPSK trandceiver system. Proceedings of the IEEE International Conference on Circuits and Systems for Communications (ICCSC).

[39] Pratt T., Allnutt J.E. (2019). Satellite Communications.

[40] Wu Y., Hu G., Jin F., Tang S.J. (2021). Multi-Objective Optimisation in Multi-QoS Routing Strategy for Software-Defined Satellite Network. Sensors.

